# A method for increasing expressivity of Gene Ontology annotations using a compositional approach

**DOI:** 10.1186/1471-2105-15-155

**Published:** 2014-05-21

**Authors:** Rachael P Huntley, Midori A Harris, Yasmin Alam-Faruque, Judith A Blake, Seth Carbon, Heiko Dietze, Emily C Dimmer, Rebecca E Foulger, David P Hill, Varsha K Khodiyar, Antonia Lock, Jane Lomax, Ruth C Lovering, Prudence Mutowo-Meullenet, Tony Sawford, Kimberly Van Auken, Valerie Wood, Christopher J Mungall

**Affiliations:** 1 European Molecular Biology Laboratory, European Bioinformatics Institute (EMBL-EBI), Wellcome Trust Genome Campus, Hinxton, Cambridge CB10 1SD, UK; 2 Department of Biochemistry, Cambridge Systems Biology Centre, University of Cambridge, Sanger Building, 80 Tennis Court Road, Cambridge CB2 1GA, UK; 3 The Jackson Laboratory, 600 Main Street, Bar Harbor, ME 04609, USA; 4 Lawrence Berkeley National Laboratory, Genomics Division, Berkeley, CA 94720, USA; 5 Centre for Cardiovascular Genetics, Institute of Cardiovascular Science, University College London, London, UK; 6 California Institute of Technology, Division of Biology 156-29, Pasadena, CA 91125, USA

**Keywords:** Gene Ontology, Functional annotation, Annotation extension, Manual curation

## Abstract

**Background:**

The Gene Ontology project integrates data about the function of gene products across a diverse range of organisms, allowing the transfer of knowledge from model organisms to humans, and enabling computational analyses for interpretation of high-throughput experimental and clinical data. The core data structure is the annotation, an association between a gene product and a term from one of the three ontologies comprising the GO. Historically, it has not been possible to provide additional information about the context of a GO term, such as the target gene or the location of a molecular function. This has limited the specificity of knowledge that can be expressed by GO annotations.

**Results:**

The GO Consortium has introduced annotation extensions that enable manually curated GO annotations to capture additional contextual details. Extensions represent effector–target relationships such as localization dependencies, substrates of protein modifiers and regulation targets of signaling pathways and transcription factors as well as spatial and temporal aspects of processes such as cell or tissue type or developmental stage. We describe the content and structure of annotation extensions, provide examples, and summarize the current usage of annotation extensions.

**Conclusions:**

The additional contextual information captured by annotation extensions improves the utility of functional annotation by representing dependencies between annotations to terms in the different ontologies of GO, external ontologies, or an organism’s gene products. These enhanced annotations can also support sophisticated queries and reasoning, and will provide curated, directional links between many gene products to support pathway and network reconstruction.

## Background

Comprehensive representation of the roles of gene products, individually and in combination, is essential to the understanding and modeling of biological systems. In addition to a gene product’s intrinsic activity, aspects of the context in which it acts, such as the gene products it acts upon, subcellular location of the activity, distribution in cell or tissue types, or temporal restrictions to a cell cycle phase or developmental stage, must be described in order to obtain a full description of its biological role.

The Gene Ontology (GO) is a bioinformatics resource that uses structured controlled vocabularies (ontologies) to describe the *molecular functions* or activities of a gene product, the *biological processes* in which a gene product is involved and the *cellular components* in which a gene product is located. Associations or ‘annotations’ can be made between ontology terms and specific genes or gene products using a variety of manual or algorithmic methods that rely upon experimental evidence or sequence similarity, for example, to support the assertion [1-4].

**Figure 1:**
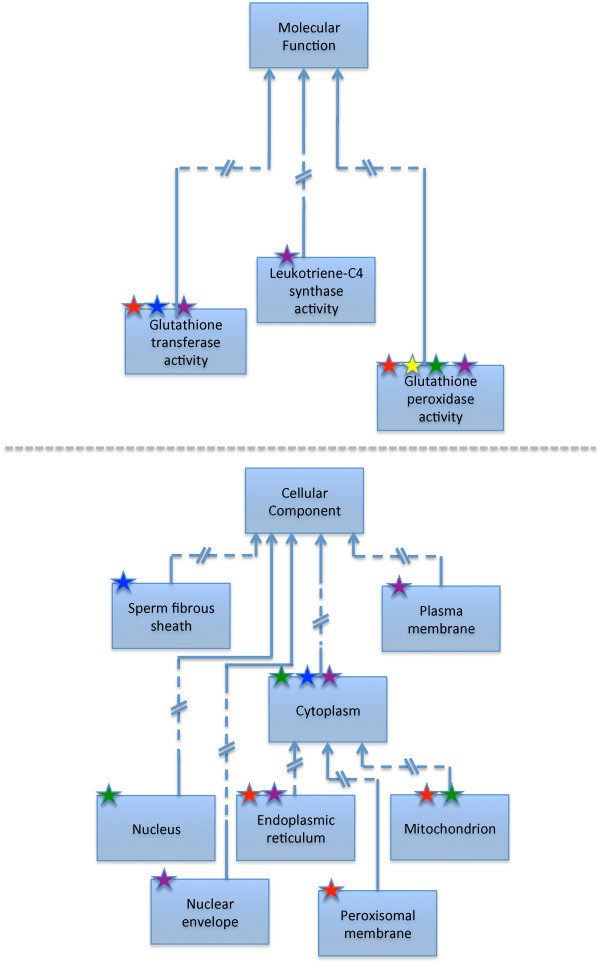
**Representation of annotations made using the core GO annotation model.** Gene products can be annotated to several GO terms, and any GO term can be used to annotate any number of gene products, but the annotations remain independent. The stars indicate an annotation of a gene product to the GO term and each colour represents a single gene product. Using this simple GO annotation model, it is not clear from the annotations shown in which Cellular Component each of the protein activities are performed. For example, microsomal glutathione S-transferase 1 is represented by the red star and can perform two activities; glutathione transferase activity and glutathione peroxidase activity. It is found in three Cellular Components, the mitochondrion, endoplasmic reticulum and peroxisomal membrane, but from these annotations the knowledge that the glutathione transferase activity is performed in the mitochondrion [7] cannot be found. For clarity not all annotations of each gene product are shown nor all terms between the specified terms and the root node.

While the ontological rigor of the GO vocabularies has been enriched over the years through the use of expressive formalisms that permit logical reasoning and interaction with external ontologies [5], the annotations themselves have, until now, remained simple declarative statements. Each GO annotation is essentially a pair, combining a single gene product with a single GO term, plus supporting metadata such as the evidence for the association [6]. Furthermore, any gene product can be associated with many GO terms, and likewise any GO term could be used to annotate any number of gene products, the annotations thus coded remain independent. The simplicity of this core GO annotation model has facilitated the population of large annotation datasets, but this simplicity has, as well, been unable to capture the interconnections between multiple annotations to multiple genes, resulting in limitations on the granularity and connectivity of information that could be captured. Figure 1 illustrates this by showing a subset of Molecular Function and Cellular Component annotations to several gene products, including microsomal glutathione transferase 1 [7]. While the annotations can describe which activities the gene products can perform, and in which components they are located, there is no way of combining this information to convey which activities are performed in which locations.

### Guidelines for pre-composing ontology terms

In the core GO annotation model, adding new terms to the ontology, or "pre-composing" terms, has traditionally captured additional biological detail. However, we have set limits on how specific terms may be differentiated from one another. For example, generally we do not add new terms for activities or processes that are identical apart from which specific genes or gene products they affect.

To illustrate this, consider the two terms ‘*regulation of transcription from RNA polymerase II promoter*’ (GO:0006357) and ‘*regulation of Sonic hedgehog transcription from RNA polymerase II promoter*’. The second term would not be added to the Biological Process ontology because the only difference between it and its parent is the target of the regulation; the core process represented by the term is mechanistically no different from analogous processes governing transcription of other genes. Although for the purpose of understanding the biology it is important to capture information about gene products that specifically regulate the transcription of Sonic hedgehog, it would not be practical to create a specific transcription regulation term in the Biological Process ontology for every regulation target in a genome.

We also want to avoid pre-composing GO terms that combine many concepts, or whose term label is very long, making it difficult for humans to easily interpret their meaning.

To enable curators to create flexible, meaningful GO annotations at the time of annotation that represent a more complete picture of gene product roles in their biological context, we have introduced **annotation extensions** to the GO annotation model. Curators can add detail to GO annotations using controlled vocabularies (either GO or external ontologies, such as Cell Type Ontology (CL) [8]; Uber Anatomy Ontology (Uberon) [9] or Plant Ontology (PO) [10]) and biological entities such as genes or their products. GO annotations with extensions thus incorporate an increased level of detail and biological integration, supporting more sophisticated querying and analysis. We have applied this model to the curation of gene products from species such as mouse, human and fission yeast and are proceeding to implement it throughout the GO Consortium.

Here we describe how annotation extensions have been incorporated into the GO annotation system, summarize the relationship types we use for extensions, and provide examples of how extensions can be displayed and applied, using a corpus of annotations we have developed.

## Results

### Extending basic annotations with relationships

We extended the core GO annotation model to accommodate annotation extensions. The annotation extension model is described formally in terms of the Web Ontology Language (OWL) in the ‘Methods’ section. Conceptually, we take existing GO terms such as ‘*protein kinase activity*’ (GO:0004672) or ‘*nucleus*’ (GO:0005634) and describe a more specific subtype through the use of one or more formal relationships to other entities (such as the protein that is the target of the kinase, or the cell type which the nucleus is a part of). This is logically equivalent to creating a new term for the subtype in the ontology.

An extended annotation is an annotation to a GO term followed by one or more relational expressions (extensions). Each relational expression is written as *Relation(Entity)*, where *Relation* is a label denoting a relationship type, and *Entity* is an identifier for a database object or ontology term. Each such expression can be thought of as refining the core GO term used. For example, the *Entity* identifier for ‘*keratinocyte*’ (CL:0000312) from the Cell Type Ontology (CL) can be combined with the *Relation ‘part_of’* to create the expression "part_of(CL:0000312)", and when combined with the GO term ‘*nucleus*’ (GO:0005634) now describes a gene product that localizes to the nucleus of a keratinocyte.

### Relations

**Figure 2:**
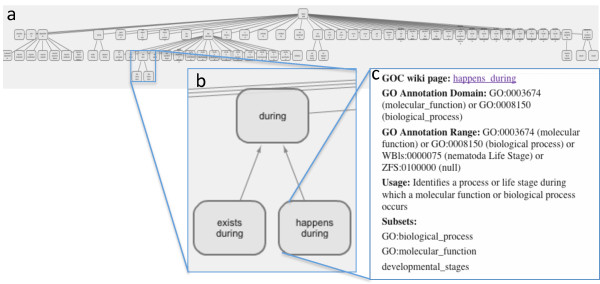
**Graphical web view of the annotation extension relations. (a)** A graphical view of the relations was created to assist curators in selecting the appropriate relation for curation [14]; **(b)** A user can zoom into a particular area of the graph; **(c)** Relations can be clicked to view information, such as which GO terms the relation can be used with and which identifiers may be used with the relation.

We created an application ontology that extends the OBO (Open Biomedical Ontologies) Relations Ontology (RO) [11] with a set of relations created explicitly for use in GO annotation extensions. These were selected and defined for practical use by iterative discussion among curators, and are collected in a file maintained in OBO format [12], and also available in OWL format [13]. To enable curators to select the appropriate relation we have created a graphical web view (Figure 2; [14]), and organized relations into subsets by entity type (for example, all relations where a chemical entity can be specified are grouped in the ‘chemical’ subset).

The set of relations used fall into two broad categories – molecular relations, which take an entity such as a gene, gene product, complex or chemical as an argument; and contextual relations, which take an entity such as a cell type, anatomy term, developmental stage or a GO term as an argument. Table 1 lists the most frequently used annotation extension relations with examples of their usage.

**Table 1:** Most commonly used relationships for annotation extension statements and examples of their usage

**Contextual relationships**	**Example (gene product; primary GO term; annotation extension)**
part_of	*C. elegans* psf-1; nucleus; part_of(WBbt:0006804 *body wall muscle cell*)
occurs_in	Mouse opsin-4; G-protein coupled photoreceptor activity; occurs_in(CL:0000740 *retinal ganglion cell*)
happens_during	*S. pombe* wis4; stress-activated MAPK cascade; happens_during(GO:0071470 *cellular response to osmotic stress*)
**Molecular relationships**	**Example (gene product; primary GO term; annotation extension)**
has_regulation_target	Human suppressor of fused homolog SUFU; negative regulation of transcription factor import into nucleus; has_regulation_target(UniProtKB:P08151 *zinc finger protein GLI1*)
has_input	*S. pombe* rlf2; protein localization to nucleus; has_input(PomBase:SPAC26H5.03 *pcf2*)
has_direct_input	Human WNK4; chloride channel inhibitor activity; has_direct_input(UniProtKB:Q7LBE3 *Solute carrier family 26 member 9*)

Molecular relations take an entity such as a gene, gene product, complex or chemical as an argument; contextual relations take an entity such as a cell type, anatomy term, developmental stage or a GO term as an argument. Entity names in italics are shown for clarity and are not part of the annotation extension format.

### Entities

Identifiers used for the entities in annotation extensions can reference GO or another ontology or database. Each identifier must have a prefix found in the GO Database Abbreviations file [15], for example "UniProtKB" (protein database) [16], "CHEBI" (chemical database) [17], "CL" (cell type ontology) [8], "Uberon" (metazoan anatomy ontology) [9], or "PO" (plant anatomy ontology) [10]. A gene product identifier used in an annotation extension, should be interpreted in the context of the primary GO term used. For example, the inclusion of the gene identifier SGD:S000004660 in the annotation extension field associated with the GO term ‘*protein phosphorylation*’ should be interpreted as "the protein product of SGD:S000004660 is phosphorylated".

### Combining multiple extensions

In this new system, a single GO annotation can have multiple relational expressions associated with it, where each expression uses a single relation and a single entity. Multiple expressions using the same relation are permitted. For example, if a gene product can carry out its activity in multiple locations or during various processes, multiple *Relation(Entity)* pairs may be added as separate annotation extensions.

To illustrate, consider a gene product that has its activity in a neuron of the hippocampus. Here it would be appropriate to make an extension combining two expressions for both the cell type (neuron) and the gross anatomical structure (hippocampus). If this gene product also had the same activity in an epithelial cell, this expression could be combined in the same annotation extension.

A description of the semantics of multiple extensions used in the annotation extension model can be found in the ‘Methods’ section.

### Annotation extensions in curation to specify molecular targets

**Figure 3:**
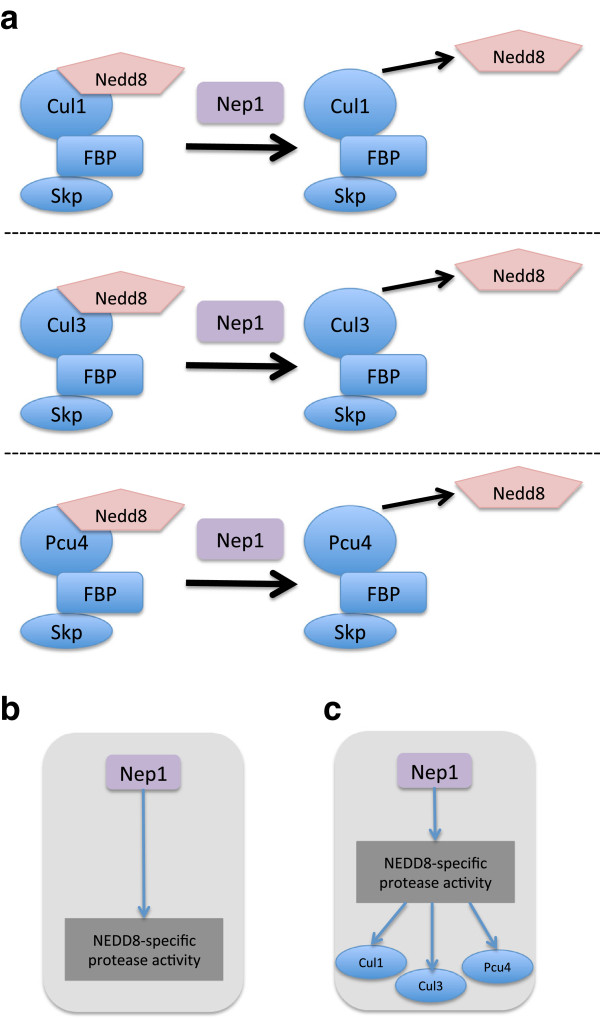
**The deneddylation activity of *S. pombe *Nep1. (a)** The experimental data reported in [18] is interpreted as: Nep1 is capable of deneddylating the cullins Cul1, Cul3 and Pcu4. **(b)** and **(c)** Graphical representation of Nep1 annotations using **(b)** the core GO annotation model and **(c)** the extended GO annotation model.

*Schizosaccharomyces pombe* protein Nep1 illustrates how annotation extensions can be used to represent the multiple targets of a gene product’s enzymatic activity. Nep1 is a protease that can deneddylate proteins modified by Nedd8 [18]. It has been shown to deneddylate three cullin proteins, Cul1, Cul3 and Pcu4 (Figure 3a). Using the core GO annotation model described above, it was not possible to record the cullins as the targets of the deneddylation activity of Nep1. The annotation would be:

*‘NEDD8-specific protease activity’* (GO:0019784) with the evidence code ‘Inferred from Mutant Phenotype’ (IMP) (Figure 3b).

Using annotation extensions the annotation can be enriched as follows: Nep1 is annotated to the term *‘NEDD8-specific protease activity’* (GO:0019784) with the evidence code IMP, and with several *Relation(Entity)* pairs specifying the gene product targets of the activity:

(See ‘Methods’ section for a description of the semantics used in the annotation extension model).

**Figure 4:**
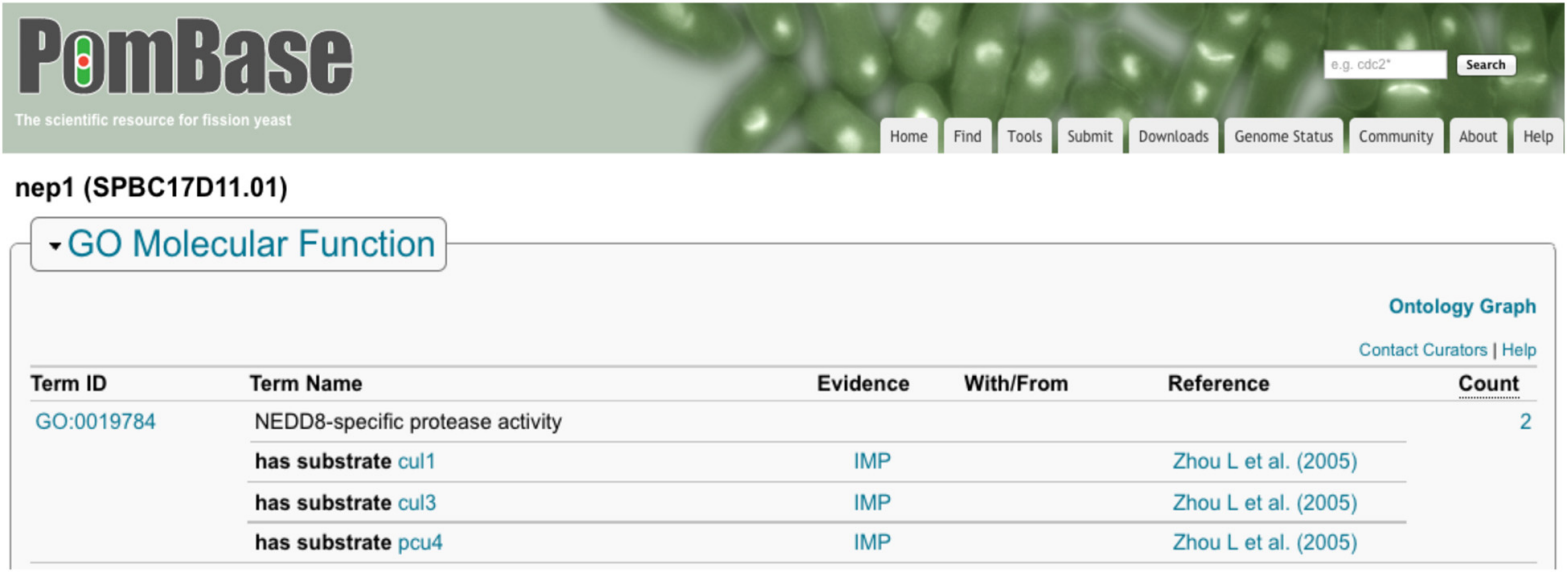
**Display of annotation extension data in PomBase for *S. pombe *Nep1 gene product.** Annotation of the observation that Nep1 deneddylates the three cullins Cul1, Cul3 and Pcu4 [18] requires one annotation with three separate expressions in the annotation extension. Note that more human-readable text has been substituted for the annotation extension relation names for display purposes in PomBase. The underlying data retain the relation names, and the mapping between relation names and display text is available on the PomBase website [19].

This annotation means that a *nep1* mutant phenotype (IMP evidence in [18]) indicates that Nep1 executes NEDD8-specific protease activity and can deneddylate Cul1 (SPAC17G6.12), Cul3 (SPAC24H6.03) and Pcu4 (SPAC3A11.08) (Figure 3c). We use the relation *has_direct_input* here with a Molecular Function term to indicate the effector–substrate relationship between the gene product and its target protein. The PomBase display of the Nep1 annotation is shown in Figure 4, note the annotation extension relation names have been translated to more human-readable text [19].

### Annotation extensions in curation to specify locational context

To illustrate how annotation extensions may be used to specify locational context, we use the example of the rat signaling complex subunit, mAKAP. mAKAP has been shown by immunocytochemical assay to be located on the nuclear envelope of cardiomyocytes [20].

With the core annotation model, we are only able to capture the cellular compartment that mAKAP is located in ‘*nuclear envelope*’ (GO:0005635) with the evidence code ‘Inferred from Direct Assay’ (IDA).

Using the annotation extension model as follows, we can capture the cellular and anatomical context of the location of mAKAP such that mAKAP is annotated to the term ‘*nuclear envelope*’ (GO:0005635) with the evidence code IDA and with two *Relation(Entity)* pairs specifying the cell and tissue locations of the nuclear envelope:

part_of(CL:0002495), part_of(UBERON:0002082)

This annotation means that a direct assay (immunocytochemical assay in [20]) has shown that rat mAKAP is located at the nuclear envelope of ‘*fetal cardiomyocytes*’ (CL:0002495) of the ‘*cardiac ventricle*’ (UBERON:0002082).

### Interconversion of core GO annotations and annotation extensions

As annotation extensions are a relatively new feature of GO curation, we have described and implemented methods that allow legacy tools (i.e. those that do not have support for extensions in their data models) to use extended annotations without loss of specificity [21]. We have also implemented reverse methods that allow the conversion of basic GO annotations to extended annotations. We informally call these methods ‘folding’ and ‘unfolding’ respectively, and these make use of the OWL formalization of the GO (see ‘Methods’ section for details). The folding operation creates a new application ontology on the fly, with each extended annotation materializing a new GO term. An OWL reasoner is used to automatically construct the graph in this new ontology. Application of this method can be seen as a stopgap to allow continued use of existing tools – the resulting application ontology, whilst logically complete, may be unwieldy for querying and browsing. The unfolding method takes annotations to existing highly specific GO terms, and replaces them with an annotation to a more basic GO term, with the equivalent additional information now expressed as extensions. Unfolding annotations is useful for reducing the complexity of GO terms when querying or browsing.

## Discussion

### Practical application of annotation extensions

Several member groups of the GO Consortium are now producing extended annotations to enrich their dataset. A summary of the numbers of extended annotations categorized by species is shown in Table 2.

**Table 2:** Extended annotations categorized by species

**Species**	**Total no. manual annotations**	**No. extended annotations**	**% extended annotations**
Mus musculus	409098	25209	6.2
Homo sapiens	219258	9042	4.1
Saccharomyces cerevisiae	53750	2713	5.0
Schizosaccharomyces pombe	29049	1902	6.5
Caenorhabditis elegans	27488	1102	4.0
Arabidopsis thaliana	101936	503	0.5
Rattus norvegicus	72280	477	0.7
Escherichia coli	11658	426	3.7
Dictyostelium discoideum	19278	228	1.2
Drosophila melanogaster	109886	214	0.2

The number of extended annotations is shown compared to the total number of manual annotations for each species. Calculated with the statistics from the UniProt-GOA database [3] on 21 November 2013.

Currently there are few applications, databases or browsers that make use of, or display, extended annotations. In addition to their inclusion in the annotation files, extended annotations are currently displayed in the GO Consortium browser, AmiGO 2 [22] and on the PomBase gene information pages [23] and there are plans to display them in UniProt-GOA’s GO browser, QuickGO [24], and on WormBase gene pages [25]. Outside of the GO Consortium, Ensembl Genomes [26] now display annotation extensions for *S. pombe* genes and these can be used for querying annotation sets in the Ensembl Fungi BioMart [27].

As extension data becomes more widely available, querying for functional information can become more sophisticated. Users of the GO will be able to query the annotations for a wealth of specific information, including connections between a gene product and other entities and processes, or the locations — at the subcellular level as well as cell and tissue types — where a gene product performs specific roles. For example, a user could query for all targets of a particular protein kinase, or compose a more specific query to find all the proteins that are involved in blood vessel remodeling during retina vasculature development in the camera-type eye. Annotation extensions capturing effector-target relationships at the cellular level will provide a rich source of directional information for regulatory network reconstruction. For instance, the *has_input* and *has_direct_input* relations can be used to connect signal transducing components of signaling pathways or to link DNA binding regulatory transcription factors with their specific target genes. The inherent directionality encoded in the extension can also be used to increase the information content of existing interaction-based networks. Annotation extensions can also assist with improving the interpretations of pathway analysis. Currently pathway analysis, which uses methods such as term enrichment and pathway topology, is hampered by the lack of functional annotation with associated contextual aspects such as cell or tissue type or dependencies on other gene products or substances [28]. GO has the potential to enable great advances in pathway analysis by providing this contextual information in annotation extensions.

### Pre- vs. post-composition of GO terms

As described above, increased specificity of GO annotations has historically been achieved by adding new, more specific ontology terms. However, new term addition cannot accommodate every detail that would be desirable to capture in GO annotations.

Using annotation extensions to increase annotation specificity is logically equivalent to creating new terms in the ontology (see ‘Methods’ section), but allows a more streamlined approach for information capture at the time of annotation. Extended annotations can be ‘folded’ to create a logical equivalent of a GO term, regardless of whether the term is included in the ontology. GO terms that are included in the ontology are said to be ‘pre-composed’, whereas the combination of terms and annotation extensions effectively ‘post-compose’ a term. It is also possible to perform the inverse and ‘unfold’ pre-composed GO terms into the equivalent extended annotation expression (see ‘Methods’ section). Whether terms are pre- or post-composed during the annotation process is thus not critical because it is possible to interconvert seamlessly between the two. Identical information can thus be captured by either of two routes, creation of a new pre-composed term or during the recording of an annotation.

Although many details captured in annotation extensions will remain outside the scope of GO terms indefinitely, GO developers will investigate systems by which annotation extensions can be automatically converted to pre-composed terms when certain criteria are met, for example where a certain number of annotations have identical extensions and the pre-composed term is in scope. The new terms will be added to the ontology using logical definitions that make them equivalent to the post-compositional annotation. Annotations made previously using post-composition can be processed to the new pre-composed terms.

In the future, maintaining a good balance between pre- and post-composition will be assisted by automated methods to reason over annotations enhanced with annotation extensions to ensure the annotations are consistently grouped by an appropriate common ancestor GO term.

### Impact on users of Gene Ontology annotation

The GO Consortium will provide annotation extension data as unfolded annotations, i.e. in the annotation files, the annotation extension will be kept in a separate field to the primary annotation. Consumers of annotation data can therefore choose to be unaffected by annotation extensions by simply ignoring the additional field. However, we do hope that users and tool developers will incorporate the extensions into their tools and workflows to provide additional specificity to their queries and tools. For example, a term enrichment tool provider might provide an option to fold the annotation extensions into pre-composed terms before a user performs term enrichment. A GO browser could be extended to include an option to search folded annotation extensions as well as regular GO terms, e.g. it would be possible to search for all gene products that are involved in epithelial cell differentiation, whether or not the cell type was curated using the specific GO term or in the annotation extension with the more general GO term ‘cell differentiation’. A basic query for a GO term will necessarily find the annotations to that term (and its child terms) with and without extensions, the user may choose whether or not to use the extension data.

We encourage users and tool developers to contact us with specific questions so we can assist them with using this data.

### Future developments

A longer-term goal of the GO Consortium is to link annotations together to fully describe the directionality and dependencies in a whole pathway or process. Although annotation extensions are not sufficient to represent complete biological pathways, they provide a valuable set of data that future work can build upon. A more expressive annotation system is now under development within the GO Consortium, which will allow curators to join annotations sourced from different publications and with different supporting evidence to describe entire pathways or sub-processes. The annotation extensions currently being captured will feed directly into the new modular annotation system [29].

## Conclusions

GO annotation extensions have been introduced to enhance the depth and utility of annotation data by capturing specific contextual information regarding a gene product’s function or location. Curators can now create, on-the-fly, complex GO annotations that describe dependencies and consequences of a gene product’s function or location more completely than was previously possible. Data curated using annotation extensions provides a repository for experimentally verified regulation targets for a wide range of gene products, including transcription factors and microRNAs, information that is currently not captured by other standardized annotation approaches. A large corpus of annotations now make use of annotation extensions, and this number is growing rapidly as groups make use of powerful curation tools such as UniProt-GOA’s Protein2GO [30]. Extensive annotation enhancement makes GO data more informative for a biologist’s understanding of a gene or process of interest, and provides additional value to the data which can be used by GO analysis tool providers to enhance the interpretation of high-throughput datasets, such as those created by next generation sequencing, transcriptomic and proteomic studies.

## Methods

### Annotation Extension Model

Annotation extensions are a means of dynamically referring to subtypes of existing GO terms, by means of sets of relation-value pairs, connected via either "and" or "or" operators (represented in GO annotation files using "," and "|", respectively).

We present a formal treatment of the GO annotation extension model and the syntax used to write extensions. This formal underpinning is necessary to clarify the semantics of annotation extensions and to enable the use of automated reasoners to perform useful computations. However, the details of the formal underpinnings can be hidden in tools used by curators and end-users, and instead presented in intuitive ways.

### Formalization

We formalize the annotation extension model in terms of Description Logics, and in particular the Web Ontology Language (OWL) [31]. The GO is already heavily axiomatized in OWL [32]. In the core GO annotation model, an annotation is an association between a gene or gene product G and an OWL Class *C. C* is restricted to be a class from one of the three sub-ontologies of the GO: Molecular Function, Biological Process, or Cellular Component. The meaning of the association varies depending on which of these three sub-ontologies are used – there are a number of ways of formalizing this in OWL, however, we do not provide details here as this is not in the scope of the extensions provided in this manuscript.

The GO annotation extension model is formally a *relaxation* of the core model, in that it allows the annotation to be to any OWL Class Expression that conforms to the following profile.

For a description of the constructs used in the above, please see the OWL2 syntax and semantics document [33]. The main language constructs used are (1) intersections, which are interpreted as set-intersection (2) existential restrictions ("some values from") which correspond to standard relationships such as those found in the GO and (3) object properties, also known as relations.

It can be seen that annotation extensions form a subset of the EL++ profile [34], which thus allows the use of fast reasoners such as Elk [35]. This is important for the GO, which contains large numbers of annotations.

One consequence of this model is that the external entities, being related, must be modelled as OWL classes rather than OWL individuals. In practice this is not a limitation, as molecular entities such as proteins are typically modelled as classes [36].

### Syntax

Annotation extensions can be expressed in a backwards and forwards compatible extension to existing exchange formats such as Gene Association Format (GAF); GAF 2.0 extends GAF 1.0 by providing an additional column (position 16) in which to write a set of relational expressions, as defined above. This column is optionally filled with a disjunctive expression conforming to the following Bachus Normal Form (BNF) grammar:

A disjunction is equivalent to multiple independent annotations each consisting of a conjunctive expression.

The conjunctive expression is translated to an OWL intersection expression whose elements are the main GO class being annotated together with all relational expressions in the conjunction. Each relational expression is translated to an OWL existential restriction ("some values from"). The Relation Symbol is translated to an Object Property from the Relations Ontology, and the ClassID is translated to an OWL class, both according to the mapping provided in the OBO format document [37]. To precisely specify the semantics of multiple extensions in output files, the annotation formats provided by the GO Consortium force the use of either the comma character (",") or the pipe character ("|") to separate each expression, where the comma indicates conjunction (AND) and the pipe indicates disjunction (OR).

For example, an annotation to the term ‘*nuclear envelope*’ (GO:0005635) with an extension field filled with:(where the CL identifier denotes *cardiomyocyte* and the Uberon identifier denotes *cardiac ventricle*) is translated to be an annotation to the OWL class expression:(where the BFO (Basic Formal Ontology) identifier denotes the *part_of* relation).

part_of(CL:0002495), part_of(UBERON:0002082)

GO_0005635 and (BFO_0000050 some CL_0002495) and (BFO_0000050 some UBERON_0002082)

These expressions can be used by OWL reasoners to return guaranteed valid and complete answers to queries such as "find all annotations to classes that are part of a cell nucleus and part of a heart".

The syntax does not allow nesting of expressions, but the use of parentheses in the grammar allows for the introduction of nesting in the future.

### Property Chains

The set of object properties used can be primitive relations (such as *part_of*, *occurs_in* or *regulates*) or relations defined via an object property chain. This effectively allows for a limited level of nesting in the annotated OWL class expression, extending the profile described above to:

For example, if a relation expression of *regulates_occurs_in*(CL:0000540) is used, this is equivalent to an OWL class expression

regulates some (occurs_in some CL_0000540)

Based on the definition of regulates_occurs_in < - > regulates o occurs_in.

These chains can be expanded in user-views – for example, AmiGO 2 will show the expression above as "regulates . occurs_in : neuron".

### Automated validation using reasoning

We use the Elk reasoner to reason over annotation class expressions in order to make sure they are logically coherent according to constraints encoded in the OWL version of the GO, the relations ontology (RO; [11]), and external ontologies. For example, an annotation to a nonsense class expression that contains **occurs_in some apoptosis** is flagged because the reasoner computes that this expression is unsatisfiable, due to the constraint that the range of occurs_in is a continuant (i.e. non-process).

We also use reasoning to automatically deepen annotations to class expressions to the Most Specific Class (MSC) in the ontology. For example, if a gene product is annotated to ‘*postsynaptic density*’ (GO:0014069) and has the extension field filled with "*part_of*(CL:0000127)", this is directly translated to the class expression **‘postsynaptic density’ and part_of some astrocyte** which is inferred to have the MSC GO:0097483 (‘*glial cell postsynaptic density*’) based on equivalence axioms in the GO [5]: **‘glial cell postsynaptic density’ EquivalentTo ‘postsynaptic density’ and part_of some ‘glial cell’** and the axiom ‘**astrocyte’ SubClassOf ‘glial cell’** inferred from the Cell Type Ontology.

These reasoner checks and deepening procedures are performed by the GO Continuous Integration server [38]. We translate Gene Association Files into OWL using OWLTools [39].

### Annotation folding and unfolding procedure

We define a process of annotation folding that takes as input the GO plus a set of supporting ontologies together with a set of extended annotations and generates as output an additional ontology plus a set of basic annotations, where the input and output are logically equivalent [21]. For each extended annotation *a* to a term *t* and extension expression *e*, we replace this with an annotation *a’* to a term *t*^A^, where *t*^A^ is added to the application ontology, with an equivalence axiom *t*^A^ EquivalentTo (*t* and *e*). A fast OWL reasoner such as Elk is used to automatically classify the application ontology. The completeness of the classification is related to the proportion of classes in the core GO ontology that have equivalence axioms.

The converse procedure of annotation unfolding takes as input the GO plus a set of supporting ontologies together with a set of basic annotations and generates as output a simplified GO plus a set of extended annotations. For each annotation *a* to a term *t*, if the term *t* has an equivalence axiom in the GO to an expression (*t’* and *e*), where *t’* is a GO term and *e* conforms to an extension expression, then replace *a* with a new annotation *a’*, where *t* is replaced by *t’* and the extension field is filled with *e*.

### Curation procedures

Annotation extensions are created as part of the manual curation process [6]. This involves biological database curators reading full text, peer-reviewed articles to obtain information about gene product functions, the processes in which they are involved and their subcellular locations [1-4]. Curators choose GO terms that describe these aspects of a gene product and assign an evidence code that is appropriate for the type of supporting experiment or statement in the paper. The GO annotations and any annotation extension information are entered into the annotating groups’ curation tool for inclusion in their database and/or display on their website. On a periodic basis, each group submits their file(s) of annotations for display on the GO Consortium website [40] and ftp site [41].

Annotation extensions are formatted as *Relation*(*Entity*) – where ‘*Entity*’ is an identifier in an ontology or database, expressed as ‘DB:ID’ – in the current GO annotation file format (GAF2.0, column 16) [42] and in the new format Gene Product Association Data (GPAD, column 11) [43]. The DB prefix must be listed in the GO Database Abbreviations collection [15].

### Data availability and resources

Annotation extensions can be represented in the two GO Consortium-supported annotation formats, GAF 2.0 [42] and GPAD [43]. These files are housed on the Gene Ontology Consortium website [40].

Annotation extension data is available in AmiGO2 [22] and for *S. pombe* genes is additionally displayed on the PomBase gene pages [44] and in the Ensembl Fungi BioMart [27].

Further documentation on annotation extensions can be found on the GO Consortium website [45].

## Abbreviations

BFO: Basic formal ontology; CHEBI: Chemical entities of biological interest; CL: Cell type ontology; GAF: Gene association file; GO: Gene ontology; GPAD: Gene product association data; IDA: Inferred from direct assay; IMP: Inferred from mutant phenotype; OBO: Open biomedical ontologies; OWL: Web ontology language; PO: Plant ontology; RO: Relations ontology; UBERON: Uber anatomy ontology.

## Competing interests

The authors declare that they have no competing interests.

## Authors’ contributions

All authors were involved in initial discussions on implementation of annotation extension and relationships. CJM, MAH, RPH, VW, ED, REF, DPH, RCL, YA-F, PM-M and JL defined the set of annotation extension relations currently in use. RPH, MAH, YA-F, ED, REF, DPH, VKK, AL, RCL, PM-M, KV-A and VW contributed annotations with extensions. TS was responsible for the development of Protein2GO to allow for curation of annotation extensions and for the graphical visualization of annotation extension relations. RPH coordinated the writing of the paper. RPH, MAH, JAB, DPH, JL, RCL, CJM, KV-A and VW contributed to the writing of the paper. SJC is responsible for the development of AmiGO2. HD worked on the OWLTools code for folding/unfolding. All authors read and approved the final manuscript.

## References

[B1] Li D Berardini TZ Muller RJ Huala E Building an efficient curation workflow for the Arabidopsis literature corpus Database (Oxford) 2012 2012 bas047 23221298 10.1093/database/bas047 PMC3515862

[B2] Drabkin HJ Blake JA Manual Gene Ontology annotation workflow at the Mouse Genome Informatics Database Database (Oxford) 2012 2012 bas045 23110975 10.1093/database/bas045 PMC3483533

[B3] Dimmer EC Huntley RP Alam-Faruque Y Sawford T O’Donovan C Martin MJ Bely B Browne P Mun Chan W Eberhardt R Gardner M Laiho K Legge D Magrane M Pichler K Poggioli D Sehra H Auchincloss A Axelsen K Blatter M-C Boutet E Braconi-Quintaje S Breuza L Bridge A Coudert E Estreicher A Famiglietti L Ferro-Rojas S Feuermann M Gos A The UniProt-GO Annotation database in 2011 Nucleic Acids Res 2011 40 D565 D570 22123736 10.1093/nar/gkr1048 PMC3245010

[B4] Pillai L Chouvarine P Tudor CO Schmidt CJ Vijay-Shanker K McCarthy FM Developing a biocuration workflow for AgBase, a non-model organism database Database (Oxford) 2012 2012 bas038 23160411 10.1093/database/bas038 PMC3500517

[B5] Mungall CJ Bada M Berardini TZ Deegan J Ireland A Harris MA Hill DP Lomax J Cross-product extensions of the Gene Ontology J Biomed Inform 2011 44 80 86 10.1016/j.jbi.2010.02.002 20152934 PMC2910209

[B6] Balakrishnan R Harris MA Huntley R Van Auken K Cherry JM A guide to best practices for Gene Ontology (GO) manual annotation Database 2013 2013 bat054 23842463 10.1093/database/bat054 PMC3706743

[B7] Johansson K Järvliden J Gogvadze V Morgenstern R Multiple roles of microsomal glutathione transferase 1 in cellular protection: a mechanistic study Free Radic Biol Med 2010 49 1638 1645 10.1016/j.freeradbiomed.2010.08.013 20727966

[B8] Meehan TF Masci AM Abdulla A Cowell LG Blake JA Mungall CJ Diehl AD Logical development of the cell ontology BMC Bioinforma 2011 12 6 10.1186/1471-2105-12-6 PMC3024222 21208450

[B9] Mungall CJ Torniai C Gkoutos GV Lewis SE Haendel MA Uberon, an integrative multi-species anatomy ontology Genome Biol 2012 13 R5 10.1186/gb-2012-13-1-r5 22293552 PMC3334586

[B10] Avraham S Tung C-W Ilic K Jaiswal P Kellogg EA McCouch S Pujar A Reiser L Rhee SY Sachs MM Schaeffer M Stein L Stevens P Vincent L Zapata F Ware D The Plant Ontology Database: a community resource for plant structure and developmental stages controlled vocabulary and annotations Nucleic Acids Res 2008 36 D449 D454 18194960 10.1093/nar/gkm908 PMC2238838

[B11] OBO Relations Ontology http://obo-relations.googlecode.com

[B12] GO Annotation Extension Relations OBO file http://purl.obolibrary.org/obo/go/extensions/gorel.obo

[B13] GO Annotation Extension Relations OWL file http://purl.obolibrary.org/obo/go/extensions/gorel.owl

[B14] GO Annotation Extension Relations graph http://www.ebi.ac.uk/QuickGO/AnnotationExtensionRelations.html

[B15] GO Database Abbreviations file http://www.geneontology.org/doc/GO.xrf_abbs

[B16] UniProt Consortium Activities at the Universal Protein Resource (UniProt) Nucleic Acids Res 2014 42 D191 D198 24253303 10.1093/nar/gkt1140 PMC3965022

[B17] Hastings J de Matos P Dekker A Ennis M Harsha B Kale N Muthukrishnan V Owen G Turner S Williams M Steinbeck C The ChEBI reference database and ontology for biologically relevant chemistry: enhancements for 2013 Nucleic Acids Res 2013 41 D456 D463 10.1093/nar/gks1146 23180789 PMC3531142

[B18] Zhou L Watts FZ Nep1, a Schizosaccharomyces pombe deneddylating enzyme Biochem J 2005 389 307 314 10.1042/BJ20041991 15769255 PMC1175107

[B19] PomBase annotation extension relation display http://www.pombase.org/documentation/gene-page-annotation-extension-relation-display

[B20] Kapiloff MS Jackson N Airhart N mAKAP and the ryanodine receptor are part of a multi-component signaling complex on the cardiomyocyte nuclear envelope J Cell Sci 2001 114 3167 3176 11590243 10.1242/jcs.114.17.3167

[B21] Annotation Extension Folding http://code.google.com/p/owltools/wiki/AnnotationExtensionFolding

[B22] AmiGO 2 http://amigo2.berkeleybop.org/cgi-bin/amigo2/amigo

[B23] Wood V Harris MA McDowall MD Rutherford K Vaughan BW Staines DM Aslett M Lock A Bähler J Kersey PJ Oliver SG PomBase: a comprehensive online resource for fission yeast Nucleic Acids Res 2012 40 D695 D699 10.1093/nar/gkr853 22039153 PMC3245111

[B24] QuickGO http://www.ebi.ac.uk/QuickGO

[B25] Yook K Harris TW Bieri T Cabunoc A Chan J Chen WJ Davis P de la Cruz N Duong A Fang R Ganesan U Grove C Howe K Kadam S Kishore R Lee R Li Y Muller H-M Nakamura C Nash B Ozersky P Paulini M Raciti D Rangarajan A Schindelman G Shi X Schwarz EM Ann Tuli M Van Auken K Wang D WormBase 2012: more genomes, more data, new website Nucleic Acids Res 2012 40 D735 D741 10.1093/nar/gkr954 22067452 PMC3245152

[B26] Kersey PJ Lawson D Birney E Derwent PS Haimel M Herrero J Keenan S Kerhornou A Koscielny G Kähäri A Kinsella RJ Kulesha E Maheswari U Megy K Nuhn M Proctor G Staines D Valentin F Vilella AJ Yates A Ensembl Genomes: extending Ensembl across the taxonomic space Nucleic Acids Res 2010 38 D563 D569 10.1093/nar/gkp871 19884133 PMC2808935

[B27] Ensembl Fungi BioMart http://fungi.ensembl.org/biomart/martview/

[B28] Khatri P Sirota M Butte AJ Ten years of pathway analysis: current approaches and outstanding challenges PLoS Comput Biol 2012 8 e1002375 10.1371/journal.pcbi.1002375 22383865 PMC3285573

[B29] Thomas P Building biological function modules from molecules to populations http://viewvc.geneontology.org/viewvc/GO-SVN/trunk/experimental/lego/docs/PThomaslego-Whitepaper-2010-03.pdf

[B30] Barrell D Dimmer E Huntley RP Binns D O’Donovan C Apweiler R The GOA database in 2009–an integrated Gene Ontology Annotation resource Nucleic Acids Res 2009 37 D396 D403 10.1093/nar/gkn803 18957448 PMC2686469

[B31] OWL Web Ontology Language http://www.w3.org/TR/owl-guide/

[B32] Hill DP Adams N Bada M Batchelor C Berardini TZ Dietze H Drabkin HJ Ennis M Foulger RE Harris MA Hastings J Kale NS de Matos P Mungall CJ Owen G Roncaglia P Steinbeck C Turner S Lomax J Dovetailing biology and chemistry: integrating the Gene Ontology with the ChEBI chemical ontology BMC Genomics 2013 14 513 10.1186/1471-2164-14-513 23895341 PMC3733925

[B33] OWL2 syntax and semantics document http://www.w3.org/TR/owl2-syntax/

[B34] OWL 2 EL profile http://www.w3.org/TR/owl2-profiles/#OWL_2_EL

[B35] ELK reasoner http://code.google.com/p/elk-reasoner/

[B36] Natale DA Arighi CN Barker WC Blake J Chang T-C Hu Z Liu H Smith B Wu CH Framework for a protein ontology BMC Bioinformatics 2007 8 Suppl 9 S1 10.1186/1471-2105-8-S9-S1 18047702 PMC2217659

[B37] OBO format document https://code.google.com/p/oboformat/

[B38] Continuous Integration of Open Biological Ontology Libraries http://bio-ontologies.knowledgeblog.org/405

[B39] OWL Tools http://code.google.com/p/owltools/wiki/OortGAFs

[B40] Gene Ontology Consortium Annotation File Download http://geneontology.org/GO.downloads.annotations.shtml

[B41] GO Consortium Gene Association ftp Downloads ftp://ftp.geneontology.org/pub/go/gene-associations/

[B42] Gene Association File Format 2.0 guide http://www.geneontology.org/GO.format.gaf-2_0.shtml

[B43] Gene Product Association Data File Format http://www.geneontology.org/GO.format.gpad.shtml

[B44] PomBase website http://www.pombase.org/

[B45] Annotation Extension documentation http://www.geneontology.org/GO.annotation.extension.shtml

